# The Effect of Antivascular Endothelial Growth Factor on the Development of Adhesion Formation in Laparotomized Rats: Experimental Study

**DOI:** 10.1155/2011/578691

**Published:** 2011-06-28

**Authors:** Murat Basbug, Nurullah Bulbuller, Cemalettin Camci, Refik Ayten, Erhan Aygen, Ibrahim Hanifi Ozercan, Zulfu Arikanoglu, Sami Akbulut

**Affiliations:** ^1^Department of General Surgery, Diyarbakir Education and Research Hospital, 21400 Diyarbakir, Turkey; ^2^Department of General Surgery, Faculty of Medicine, Firat University, 23100 Elazig, Turkey; ^3^Department of Medical Pathology, Faculty of Medicine, Firat University, 23100 Elazig, Turkey

## Abstract

*Aims.* This study determined the effects of a single dose of bevacizumab, an antiangiogenic recombinant monoclonal antibody that specifically targets vascular endothelial growth factor (VEGF), on adhesion formation in the rat cecal abrasion model. *Methodology.* Thirty female Wistar albino rats (200–224 g) were divided into three groups. All rats underwent laparotomy at which time cecal wall abrasion and abdominal wall injuries were induced. Group I (control) underwent only the abrasion procedure; Groups II and III received saline or bevacizumab intraperitoneally, respectively, following the abrasion. The rats were killed on postoperative day 7, and the severity of adhesions was evaluated, together with histopathological fibrosis parameters and immunohistochemical staining to identify the VEGF receptor. *Results.* The mean adhesion severity score in Groups I–III was 2.5 ± 0.52, 2.4 ± 0.69, and 0.7 ± 0.82, respectively; the score in Group III was significantly lower than that in Groups I (*P* < 0.001) and II (*P* < 0.001). In the histopathological evaluation, the mean fibrosis score in Group III was significantly lower that the scores in Groups I (*P* < 0.001) and II (*P* < 0.001). VEGF staining of the adhesion areas in Group III was significantly lower than that in Groups I (*P* < 0.001) and II (*P* < 0.001). *Conclusion*. Bevacizumab decreases adhesion formation following laparotomy in rats by blocking VEGF receptor occupancy.

## 1. Introduction

Postoperative adhesion formation is a major clinical problem in patients who undergo abdominal surgery [1, 2]. Peritoneal adhesions are defined as pathological fibrotic bands that develop between any surfaces in the peritoneal cavity [2]. These adhesions can be induced by infection, inflammation, ischemia, and surgical injury and are the leading cause of pelvic pain, infertility, and bowel obstruction. The mechanisms underlying the predisposition to form adhesions and their site specificity are unknown [2–4]. Intra-abdominal adhesions are believed to develop as a result of ischemia and trauma to the serosal surface of bowel or peritoneum [4–6]. After peritoneal injury, vascular permeability is increased in vessels supplying the damaged area; this is followed by an exudation of inflammatory cells ultimately leading to the formation of fibrin matrix, which connects two injured peritoneal surfaces forming fibrin bands [6, 7]. 

Following fibrin band formation, fibrinolysis breaks the bands into smaller molecules as fibrin degradation products [7, 8]. If the fibrinolysis system is depressed, the adhesions are not lysed completely and the fibrin bands persist [8]. The tissue forming the adhesions is a mixture of macrophages, red blood cells, fibroblasts, nerve fibers, and small vascular channels of endothelial cells. Macrophages play an important role in this condition as they synthesize and release growth factor, which is mitogenic, chemotactic, and angiogenic [1, 9].

Angiogenesis, the process of developing new blood vessels, is a fundamental process in inflammation and wound repair. Angiogenesis is turned on or off by regulatory factors located in the extracellular matrix, which acts as a reservoir of factors that can be released after wounding or under other physiological conditions [10]. Human peritoneal capillaries and arteriole endothelial cells express vascular endothelial growth factor (VEGF) and angiogenic factors that regulate the proteolytic enzymes and their inhibitors. 

Since VEGF plays a key role in coagulation, fibrinolytic, and angiogenic activities, it is considered a critical cytokine in the development of peritoneal adhesions [1, 10, 11]. We hypothesized that the angiogenesis inhibitor bevacizumab can reduce peritoneal adhesions by increasing VEGF levels and investigated the effects of bevacizumab on intraperitoneal adhesions.

## 2. Materials and Methods

### 2.1. Protocol

This study was conducted in the Experimental Animal Raising and Research Laboratory of Firat University, Faculty of Medicine, Elazig, Turkey, after the approval of the local ethics committee. All experimental manipulations were in accordance with the National Institutes of Health Guide for the Care and Use of Laboratory Animals.

### 2.2. Animals

Thirty female Wistar albino rats (11–12 weeks of age, weighing 200–224 g) were acclimatized for 1 week before the experiments. The animals were kept in individual cages, housed at constant room temperature, and given standard rat chow. Only water was provided in the 12 h preceding the experiments.

### 2.3. Experimental Groups

The rats were divided randomly into three groups by block randomization using Random Allocation Software ver. 1.00. The researchers were blinded to the treatment, saline, and control groups. In Group I (control), abrasion only was performed. In Group II, abrasion was performed and 0.9% NaCl was administered intraperitoneally. In Group III, abrasion was performed and 2.5 mg/kg bevacizumab (Avastin, 25 mg/mL, Roche, Basel, Switzerland) was administered intraperitoneally.

### 2.4. Experimental Design

All rats were anesthetized with a combination of 5 mg/kg intramuscular xylazine (Bayer, Istanbul, Turkey) and 30 mg/kg ketamine hydrochloride (Parke-Davis, Istanbul, Turkey). All animals breathed spontaneously throughout the procedures. The mid-abdominal area was shaved and prepared with povidone-iodine as antiseptic. A 3 cm midline incision was made and the cecum was exteriorized. A 1-2 cm^2^ area of the cecum was brushed eight to ten times with a gauze bandage, and then a 1 cm^2^ peritoneal injury on the right abdominal wall opposite to the cecum was also produced by brushing. The abdominal incision was closed with continuous 3-0 silk sutures. Only water was given to all of the animals on the first postoperative day; standard rat chow and water were provided on the succeeding days.

On postoperative day 7, the rats were anesthetized as previously described and a repeat laparotomy was performed with a reversed U-shaped incision of the anterior abdominal wall, which was retracted caudally to provide maximal exposure, without damaging the area in which the abrasion had been performed.

### 2.5. Adhesion Assessment and the Adhesion Severity Score

**Figure 1:**
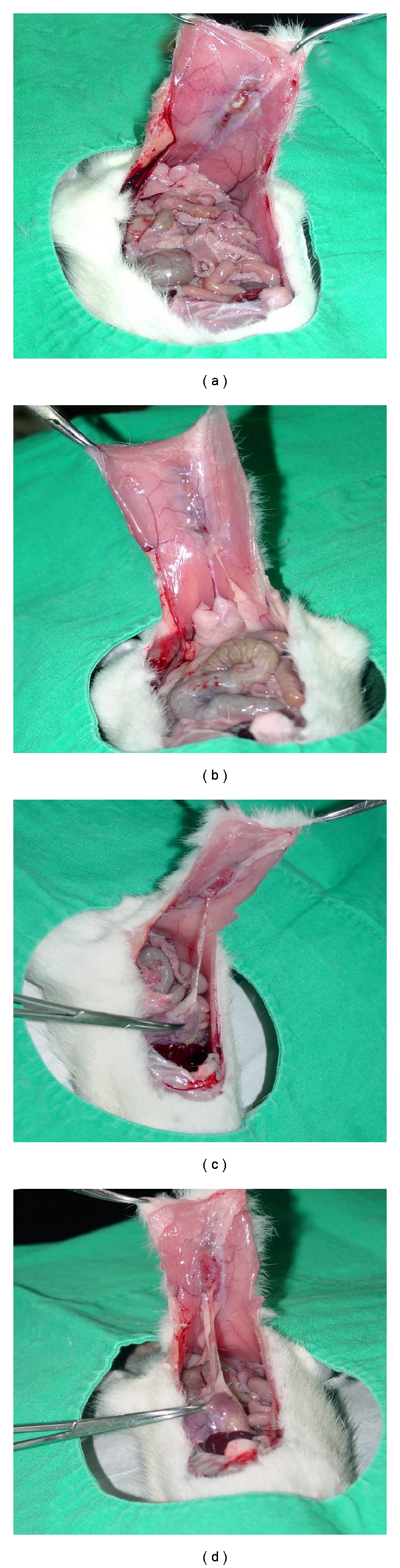
Appearance of peritoneal adhesions in rats. Grade 0 (a), I (b), II (c), and III (d) adhesions.

Adhesions were assessed between organs and the abdominal wall and among the organs themselves. Two surgeons blinded to the study (not members of the surgical team) scored the adhesions separately, and a consensus score was obtained for each rat. The type of adhesions was scored according to the method of Evans et al. [12], in which Grade 0 indicates no adhesions, Grade I indicates adhesions separating spontaneously, Grade II indicated adhesions separating by traction, and Grade III indicates adhesions separating with sharp dissection (Table 1, Figures 1(a)–1(d)).

### 2.6. Fibrosis Score

The adhesions were excised with the adhesion surfaces, and the resected adhesion specimens were fixed in formaldehyde. After dehydration, they were embedded in paraffin. Then, 5 mm cross-sections were prepared, stained with hematoxylin and eosin (H&E), and evaluated under a light microscope at a magnification of ×100. The adhesions were categorized as histopathological Grades 0–III based on the presence and extent of fibrosis [1, 4–6]. All evaluations were performed by an experienced pathologist who was blinded to the groups. Grade 0 was defined as no fibrosis, Grade I as slight fibrosis, Grade II as intermediate fibrosis, and Grade III as severe fibrosis (Table 2).

### 2.7. Immunohistochemical (IHC) Staining Procedure

**Figure 2:**
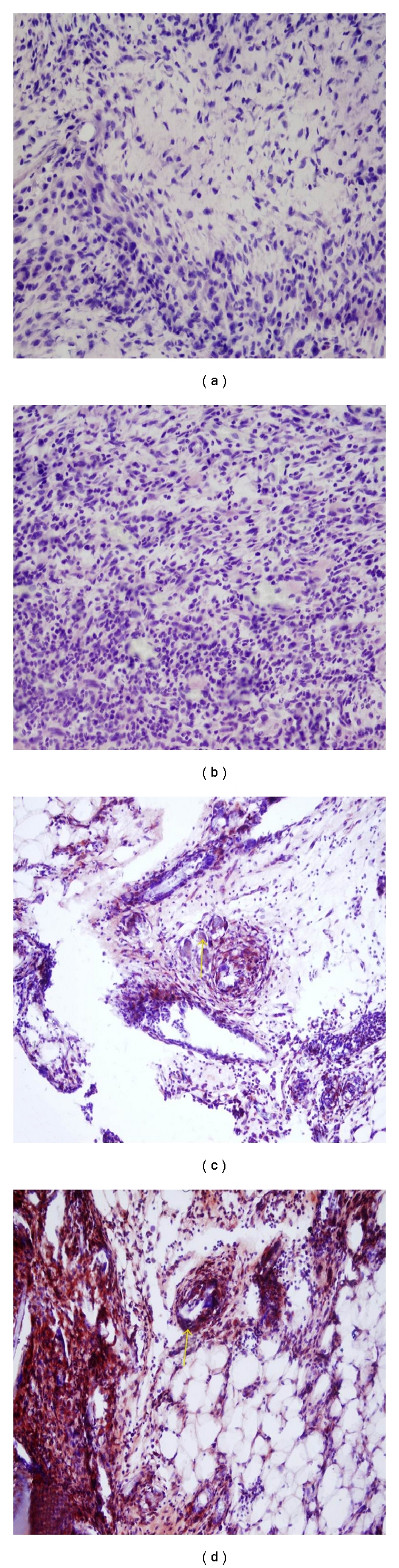
Immunohistochemistry for VEGF. No staining (a), suspected staining (b), moderate staining (c), and strong staining (d).

The adhesions were excised with the adhesion surfaces. The adhesion tissue was placed in 10% formaldehyde for both VEGF (NeoMarkers, ready to use; Neomarkers Inc., Fremont, CA, USA) receptor level measurements and IHC analysis. After few hours in fixative, the biopsy specimens were embedded in paraffin, and 5 *μ*m slices were cut and placed on a microscope slide. The tissue was stained with IHC stain used to identify the VEGF receptor. All evaluations were performed by an experienced pathologist who was blinded to the groups. The IHC staining severity and density of VEGF antibodies were evaluated in the areas where the stained cells were found in the adhesion tissues. The results were evaluated as follows: 0: no staining (negative), 1 = suspected, 2: mild, 3: moderate, and 4: strongly positive (Figures 2(a)–2(d)) [9].

### 2.8. Primary and Secondary Endpoints

The primary endpoint of this experimental study was the macroscopic adhesion score, which is the sum of the adhesion severity grading. The secondary endpoint was the microscopic fbrosis grading, extracted from the adhesion model area.

### 2.9. Statistical Analysis

The data were analyzed using SPSS 17.0 for Windows (SPSS Inc., Chicago, Il, USA). Percentages were compared with Student's* t*-test, and the Pearson Chi-square test was used for nonparametric values. The *P* values given are 2-sided; *P* < 0.05 was considered to be the limit of significance.

## 3. Results

A total of 30 rats were operated. There was no wound dehiscence; three animals developed an incision hernia: 2 in Group II and one in Group III. 

### 3.1. Adhesion Severity Score

**Figure 3:**
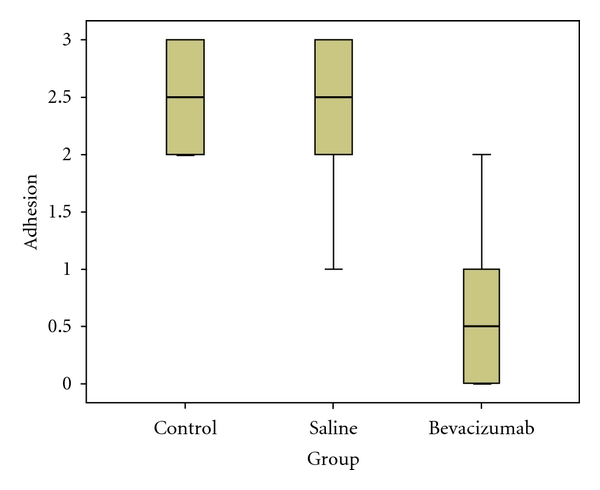
The bevacizumab group had a significantly lower adhesion grades.

Statistical comparison showed that the adhesion severity score in the bevacizumab group (Group III) differed significantly from the scores in Groups I (*P* < 0.001) and II (*P* < 0.001), while no difference was observed between Groups I and II (*P* = 0.72). The adhesion scores of the three groups and statistical analysis are summarized in Table 3. The statistical differences among all groups are also shown in Figure 3.

### 3.2. Histopathological Fibrosis Score

**Figure 4:**
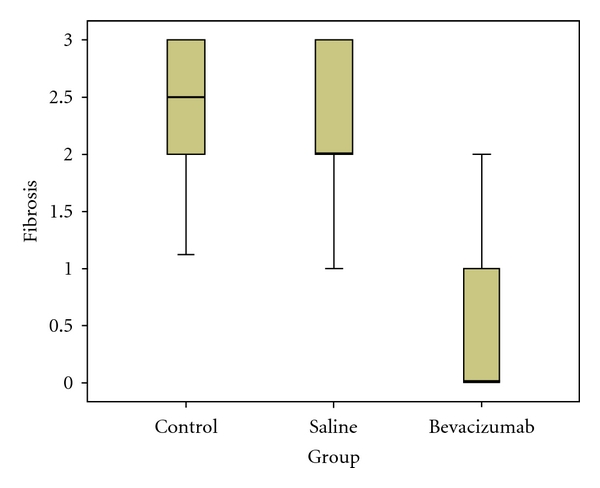
The bevacizumab group had a significantly lower fibrosis scores.

The fibrosis score in Group III was significantly less than that in Groups I (*P* < 0.001) and II (*P* < 0.001), while the fibrosis score did not differ significantly between Groups I and II (*P* = 0.55). The fibrosis scores and the statistical analysis are summarized in Table 4. The statistical differences among all groups are also shown in Figure 4.

### 3.3. Immunohistochemical Staining for VEGF

**Figure 5:**
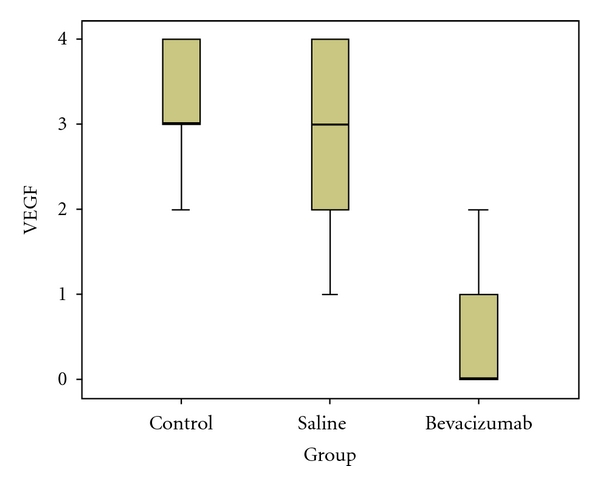
The bevacizumab group had a significantly lower staining with VEGF immunohistochemical stain.

The VEGF staining of the adhesion areas in Group III was significantly lower than that in Groups I (*P* < 0.001) and II (*P* < 0.001), while no significant difference was observed between Groups I and II (*P* = 0.27). The VEGF staining scores and the statistical analysis are summarized in Table 5. The statistical differences among all groups are also shown in Figure 5.

## 4. Discussion

Abdominal and pelvic adhesions are a major cause of morbidity, resulting in abdominal and pelvic pain, infertility, and small bowel obstruction. They are responsible for 30–41% of all intestinal obstructions [13]. Furthermore, pelvic adhesions resulting in mechanical blockage of the fallopian tubes are an important cause of infertility [14, 15]. Despite technological advances, postoperative peritoneal adhesions continue to constitute significant problems and remain a source of frustration for patients who have undergone laparotomy [16].

Various models have been developed to induce postoperative peritoneal adhesions experimentally, including local peritoneum excision, ischemic damage, the introduction of foreign objects into the peritoneal cavity, thermal damage, and bacterial contamination [4]. Any manipulation performed by the hands or surgical instruments during laparotomy constitutes mechanical trauma, which is the most frequent cause of postoperative peritoneal adhesions [4, 5]. We used a cecal abrasion model because it mimics the mechanical trauma that occurs during laparotomy.

Peritoneal adhesions are actually the result of normal wound healing, and postoperative peritoneal adhesions are seen most commonly within 7 to 15 days after surgery. Four similar, previously published studies were performed with species-specific antibodies to VEGF; in these studies, the test period (relaparotomy) was restricted to 7 and 30 days [1, 3, 9]. Our study was performed with a humanized antibody, in a species where abundant literature suggests a similarity in effect of bevacizumab in rats and humans. Our follow-up period was 7 days, and the adhesion maturation process can be affected by the reabsorbed circulating bevacizumab since it remains in circulation up to 6 weeks [1].

The search for an effective antiadhesion device has been continuing for decades. Several methods, materials, and agents have been assessed for their ability to prevent intra-abdominal peritoneal adhesions, including various surgical procedures, minimally invasive and laparoscopic techniques, pharmacological agents that target fibrin formation, and liquids, gels, and solids that can form a mechanical barrier between mesothelial surfaces [3–6].

Many animal and clinical studies have tested a variety agent to prevent intra-abdominal adhesion formation. These agents include dextran, honey, resveratrol, hyaluronic acid corticosteroid, saline, recombinant tissue plasminogen activators, aprotinin, atorvastatin, octreotide, heparin, nonsteroidal inflammatory drugs, tenoxicam, mitomycin, sildenafil, vitamin E, melatonin, and *β*-glucan [1, 3–7]. The intra-abdominal administration of antiadhesive barriers, such as a bioresorbable membrane consisting of sodium hyaluronate, polyethylene glycol, fibrin sealant, oxidized regenerated cellulose, expanded polytetrafluoroethylene, and carboxymethylcellulose, may reduce postoperative adhesions, as demonstrated by some animal models and clinical studies [1, 4–6]. Some of these agents have been shown to reduce the number and quality of adhesions, but none are universally effective and their modes of action are poorly understood [8, 17].

This study investigated the effect of bevacizumab, a monoclonal antibody against VEGF, in preventing peritoneal adhesions. The adhesions were graded using the method of Evans et al. [12]. The intensity of peritoneal adhesions was reduced dramatically in the bevacizumab group compared to the controls and 0.9% NaCl group (*P* < 0.001).

Intraperitoneal adhesion formation is a complex process that involves multiple factors, including the proliferation of blood cells and matrix components and angiogenesis. Theoretically, angiogenesis should play an important role in the development of intra-abdominal adhesions because the extent of early neovascularization correlates with adhesion formation.

The mesothelial and vascular endothelial cells in the peritoneal blood vessels, which supply peritoneal adhesions, express both VEGF and fibroblast growth factor-2 (FGF-2), indicating a role for these cytokines in mediating peritoneal angiogenesis during adhesion formation [9]. 

Human peritoneal capillaries and arteriolar endothelial cells express VEGF and other angiogenic factors that may regulate proteolytic enzymes and their inhibitors. VEGF is a critical cytokine in the development of peritoneal adhesions, and it has an essential role in the induction of angiogenesis; it is also an endothelial cell-specific mitogen [10, 18–20].

VEGF is a potent, angiogenic cytokine that is involved in the formation of adhesions; perhaps its role is to induce the development of new blood vessels supplying areas of tissue damage/injury induced by surgery [21]. It is also involved directly in tissue restoration, including the early inflammatory responses and wound repair and remodeling via fibroblast function [22]. The central role of VEGF in facilitating the deposition of the fibrin-rich matrix necessary for subsequent cellular migration and proliferation would seem to make it a key agent in the formation of peritoneal adhesions [22]. Cahill et al. [23] showed that it is involved centrally in the early pathogenesis of postoperative adhesion formation and that mast cells seem to be responsible for the early surge in peritoneal VEGF levels after an operation.

Bevacizumab is a recombinant humanized monoclonal antibody that binds all biologically active isoforms of VEGF and inhibits binding of this cytokine to its receptors: VEGFR-1 and -2 [24–27]. It neutralizes the biological effects of VEGF, including endothelial cell mitogenesis, vascular permeability enhancement, and the promotion of angiogenesis. In mouse models, the administration of anti-VEGF antibodies was shown to block the growth of human tumor xenografts and reduce the size and number of metastases [18, 28].

VEGF seems to have important roles in the early formation of intra-abdominal postoperative adhesions. It is released into the peritoneal cavity from the injured vasculature after surgery. Bevacizumab neutralizes VEGF and blocks its signal transduction through both VEGFR-1 and VEGFR-2, as demonstrated by the inhibition of VEGF-induced cell proliferation and the modulation of peritoneal adhesions by neutralizing antibodies [29, 30]. The role of selective, antiangiogenic inhibitors in the treatment of neoplastic processes may be expanded to include antiadhesion strategies [29].

In our model, following the administration of bevacizumab, VEGF receptor levels and angiogenesis decreased in the excised adhesion surfaces and fibrin tissue, as shown in the histopathological examination. The application of bevacizumab reduced angiogenesis, which may have accompanied the reduction in adhesion formation. Bevacizumab reduced the VEGF receptor count in the injured tissue. Therefore, bevacizumab lowers the formation of adhesions by binding and blocking VEGF receptors. This result may open new horizons for this drug, which is currently prescribed for anticancer purposes, in preventing adhesion formation following laparotomy in high-risk patients.

We conclude that bevacizumab prevented postoperative adhesion formation experimentally. However, additional research and clinical trials are needed to investigate and validate its long-term effects and to establish a safe protocol for its use.

## Figures and Tables

**Table 1:** Definitions of the grades of peritoneal adhesions according to Evans.

Grade	Definitions of severity grades of the peritoneal adhesions according to Evans model
0	No adhesions
1	Thin, avascular, spontaneously separating adhesions
2	Limited vascularization, adhesions separating by traction
3	Good vascularization, adhesions separating by dissection

**Table 2:** Definitions of the histopathological fibrosis score.

Grade	Definitions of the histopathological fibrosis scoring
0	No fibrosis (no fibroblast and/or collagen fiber)
1	Slight fibrosis—few fibroblast and/or collagen fibers
2	Median fibrosis (more fibroblast and/or collagen fibers)
3	Severe fibrosis (lots of fibroblasts and/or collagen fibers)

**Table 3:** Macroscopic adhesion severity grades and mean group scores.

Groups	Adhesion severity score				
	Grade 0	Grade 1	Grade 2	Grade 3	Mean ± SD
I	0	0	5	5	2.5 ± 0.52
II	0	1	4	5	2.4 ± 0.69
III	5	3	2	0	0.7 ± 0.82

*I versus II:* *P* = 0.72*; I versus III; P* < 0.001*; II versus III; P* < 0.001.

**Table 4:** Microscopic histopathological fibrosis severity grades and mean group scores.

	Fibrosis score				
Groups	Grade 0	Grade 1	Grade 2	Grade 3	Mean ± SD
I	0	1	4	5	2.4 ± 0.69
II	0	2	4	4	2.2 ± 0.78
III	6	3	1	0	0.5 ± 0.70

*I versus II: P* = 0.55; *I versus III:*; *P* < 0.001; *I versus III: P* ≤ 0.001.

**Table 5:** The severity of immunohistochemical staining with VEGF antibody.

	VEGF immunohistochemical staining					
Groups	Negative (=0)	Suspected (=1)	Mild (=2)	Moderate (=3)	Strongly (=4)	Mean ± SD
I	0	0	2	4	4	3.2 ± 0.78
II	0	2	2	3	3	2.7 ± 1.16
III	6	3	1	0	0	0.5 ± 0.70

*I verus II: P* = 0.27*; I versus III; P* < 0.001*; II versus III:* *P* < 0.001.

## Abbreviations

VEGF: Vascular endothelial growth factor
FGF-2: Fibroblast growth factor 2.
